# Calculate of withdrawal times of clenbuterol in goats to obtain safe times of slaughter

**DOI:** 10.14202/vetworld.2018.731-738

**Published:** 2018-06-01

**Authors:** Lazuardi Mochamad, Bambang Hermanto, T. I. Restiadi

**Affiliations:** 1 Department of Basic Science, Veterinary Pharmacy Subdivision, Faculty of Veterinary Medicine, Universitas Airlangga, Surabaya, Indonesia; 2 Department of Pharmacology, Faculty of Medicine, Universitas Airlangga, Surabaya, Indonesia; 3 Department of Reproduction, Faculty of Veterinary Medicine, Airlangga University, Surabaya, Indonesia

## Abstract

**Background and Aim:**

Clenbuterol as a β_2_-agonist drug was investigated according to the concentration of the drug available in the bodies of goats and according to the level of sensitivity of the instruments used for detection. The objective of the current study was to determine withdrawal times after giving a therapeutic dose that resulted in safe slaughters.

**Materials and Methods:**

Five healthy male goats with a mean body weight of 20.64 kg were treated with a single dose of 5.10^−3^ mg^/^kg in the BW onto jugular vein. Whole blood samples of approximately 5 mL were taken in a time series at 5, 30, 60, 90, 150, 210, 270, 390, 510, 630, and 750 min. At 24 h posttreatment, all subjects were sacrificed, and 300 g samples of the liver were obtained. The plasma concentration and liver residue of the drug were observed by reverse-phase high-performance liquid chromatography.

**Results:**

The drug reached a maximum concentration of 19.233±0.331 µg/mL at 5 min, and the elimination half-life was at 173.25 min. The limit detection was obtained at 0.053 µg/mL. A one-way analysis of variance between all goats showed that elimination of the clenbuterol in their bodies was similar (p=1.00), with a withdrawal time of 1,479.326 min and no residues in the liver (p<0.05).

**Conclusion:**

Safe times for slaughter were determined to be at 2 days, 13 h, and 12 min as the 2^nd^ safety factor (SF) time and 3 days, 1 h, and 58 min as the 3^rd^ SF time with the liver organ free from residue.

elimination half-life, new method for calculating withdrawal time, prescriptions for obtained β_2_-agonist, residues in liver.

## Introduction

One health system for human health concepts involved using all aspects that can drive the quality of health improvement. Food is a major aspect that can directly affect human health, especially the presence or absence of harmful chemical residues including veterinary drugs. We know that a β_2_-agonist is an active substance and it is commonly used only as a veterinary therapeutic agent. However, some users have abused the β_2_-agonist as a veterinary drug using it as a growth-promotion agent [1]. For example, ractopamine is a veterinary drug promoted for use as a supplement for growth promotion [2]. Some countries in Europe and South-East Asia have identified residues from selected veterinary drugs and have banned all residues veterinary drug products such as ractopamine [3,4]. Clenbuterol has been sold as a β_2_-agonist agent, not for growth promotion but for therapy of diseases in large polygastric animal species, for example, goats, cows, and cattle. Clenbuterol has been legally used in horses, a large monogastric animal species. The hazard of using clenbuterol in large polygastric animals needs to be determined to assess the risk of drug residues in the product, which has implications for human health. Problems with the use of clenbuterol in animals intended for human consumption, i.e., the withdrawal time, suggest safety factors (SFs) in the consumption of veterinary products, i.e., meat, eggs, and milk, after the administration of a β_2_-agonist as a therapeutic agent [5]. The withdrawal time for clenbuterol in large polygastric animals has not been determined. That timing is very important because it can be used to predict when animals can be slaughtered after receiving clenbuterol [6]. Goats are a polygastric species and are one of the consumption animals in which SF levels have not been determined yet [7].

Focusing on residues of clenbuterol, it is known that residues of the active substance in meat are not destroyed at 100°C. Due to its physicochemical properties; clenbuterol does not melt at a temperature of 100°C. Some clenbuterol, especially the 1-(4-amino-3,5-dichlorophenyl)-R, is known for its difficulty in being metabolized by substitution or conjugation and having a melting point >100°C. Compounds of 1-(4-amino-3,5-dichlorophenyl)-R are very stable. As a consequence of their physical and chemical characteristics, the compound was not easy to metabolize. Characterized compounds of 1-(4-amino-3,5-dichlorophenyl)-R were at risk to result in metabolite residues [8-10].

Based on the problems identified above, we sought to identify the withdrawal level SF of clenbuterol when treating a local breed of goats. For clarification of the residues in the organ, we also check liver levels.

## Materials and Methods

### Ethical approval

In order to guarantee a safe, correct and carefully handling of the goats, authors proceeded according to specification of International Ethics Guidelines (Internal Ethical Committee From Faculty of Veterinary Medicine, Universitas Airlangga by Clearance Recommended No. 604-KE, August 11^th^, 2016).

### Animals and experimental design

The research was carried out in facilities at the Institute for Tropical Diseases Center at the Universitas Airlangga and Center for Research, Applications and Services Veterinary Pharmacy Laboratory at Faculty of Veterinary Pharmacy in Universitas Airlangga, Surabaya, Indonesia. The room temperature of the laboratory ranged from 23°C to 25°C at a relative indoor humidity of 48%-50%. This study was conducted in compliance with the animal ethics clearance from the Faculty of Veterinary Medicine, Universitas Airlangga (Authorization register 604 KE). The goats were identified as Etawah goats, a local Indonesian breed, with the following characteristics: Male, 18 months of age, and in a healthy condition (average body weight of 20-21 kg or at mean 20.64±0.47 kg) under oversight veterinary practices. The animals adapted for 2 weeks to housing in a hygienic stable. The experimental design included a time series with observations was made at 5, 30, 60, 90, 150, 210, 270, 390, 510, 630, and 750 min posttreatment with the elimination half-life (T_1/2β_) through calculating the elimination rate constant (K_el_) of the drugs from 5 goats [11]. The time of 5 min after treatment, as referred by Lazuardi [12], explained that 0-5 min of i.v. administration was representative of leg time. For postmortem residue analysis of the drug in organs, 300 g of liver from each goat were explored after necropsy of the cadaver at 24 h posttreatment.

### Analysis of clenbuterol and the validation method

Pure clenbuterol as a reference material dissolved in non-matrix biologic was determined by spectrophotometry at a specific wavelength using a Shimadzu ultraviolet (UV)-1800 through scanning in the wavelength range 200.0 nm-245.0 nm. Clenbuterol in plasma was measured with high-performance liquid chromatography (HPLC) using a Shimadzu CBM-20A Communication Bus Module for interaction with a photodiode array detector UV-visible M20A, in which a LiChrospher^®^ 100 RP-18 column was a perfect fit. The following settings were applied with the isocratic method: 0.5 mL/min flow rate and 300 kgf/c maximum pump at a wavelength of 223.0 nm. All chemicals were of high-purity grade, and clenbuterol was a certified reference material of the European Pharmacopoeia level CAS No. 21898-19-1. The mobile phase of the fraction used acetonitrile: water (30:70) containing 0.10% phosphoric acid at pH 3.8 [13]. The analyte solvent was acetonitrile analytic grade: water for chromatography at a composition fraction 30%:70% containing 0.10% phosphoric acid adjusted in an acid condition with pH ranging from pH 3.8 to pH 4.0. Artificial plasma of the goats free from drug residues was obtained from the Central of Veterinary Pharmaceutical, Directorate General of Livestock Services, Republic Indonesia at Ahmad Yani Rd Surabaya, Indonesia. Liver organs free from the residue of the drug as medium matrix biology were obtained from a goat slaughterhouse that belonged to the Surabaya Government in Pegirian Rd, Surabaya, Indonesia.

Validation methods started from the 4^th^ point test as follows: (a) Linearity test to determine the relationship between concentration of analytes to the response detector, (b) intraday precision test, (c) accuration test by assessed recovery levels, and (d) sensitivity test using the method limit of detection (LOD) and limit of quantification (LOQ). Points a, b, and c of the validation method were using triplo iteration between concentrations 0.25 µg/mL and 1.00 µg/mL using the artificial plasma from the goats and liver organs free from residue of the drug as medium matrix biology [14]. The sensitivity tests used mobile phase solvent of the HPLC system from pH 3.8 to pH 4.00.

### Determination of withdrawal time and analysis residue from livers

Clenbuterol injections of 50 mL containing clenbuterol-HCl 0.02651 mg/mL of clenbuterol-HCl were obtained from Grovet BV, the Netherlands with a recommended dose of 5.10^−3 mg^ clenbuterol-HCl/kg body by route of intravenous. Blood samples (5 mL) were collected in glass heparin vacutainer tubes; then the plasma was separated from the whole blood through centrifuging at 1300× *g* and kept in a refrigerator at −20°C until the preparations. At 24 h posttreatment, all subjects were sacrificed, and necropsies were performed to obtain liver organs. The liver organ was placed in an area 2 cm from the portal vein and marked as code liver-1 to code liver-5 as specimens from subjects no 1 to 5 then directly kept in an inactive bottle (dark glass sample bottle) in a cold room at 4°C until preparation [15].

The LOQ was assessed from the lowest concentration of an analyte in a sample that could be determined from the chromatogram area. The determination of low concentrations of analyte required that the highest ratio of the analyte peak versus the noise peak was ≥5. The mobile phase eluent was injected 3 times, and analysis was obtained by visual evaluation of Np-p using the width of the 20^th^ analyte on the chromatogram. The blank was injected 3 times to obtain the standard deviation of the blank signal (S_B_). Next, two other concentrations, an upper and lower concentration of the analyte, were injected to obtain the sensitivity slope (S). The last step was to calculate the LOD and LOQ limit from the equation below with k=3 as the constant. The LOQ was calculated from 3 times the LOD, with 
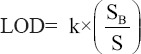
 as referenced from new techniques reported in the previous studies [14-17].

Sample preparation from plasma included the following process: First, the solid-phase extraction (SPE) column SOLA HRP 10 mg/mL cartridge was activated by adding 1 mL of methanol continuously, 1 mL water, and then 1 mL of sample. Second, the SPE column was dried in a warm temperature in an incubator for 30 min before beginning the elution. Finally, the SPE column was used to elute 1 mL of the mobile phase, and the eluent containing the active substance was collected and then dried, yielding a sample ready for use. The standard curve in the plasma matrix for the concentration versus the response detector was analyzed by the regression-correlation method with Minitab 18.0 (significant at 0.05). For determination of the withdrawal time, the following equation of 1 at SF 2 or 3 and the data maximum concentration (C_0_) at the first sampling, T_½β_ were used at 0.693/K_el_. The equation notation indicated that the Ln R accumulation factor at 24 h was approximately 1.306±0.05 and the C_lim_ was the LOQ [18]. The K_el_ was calculated from the slope of the exponential equation from the regression concentration versus time.


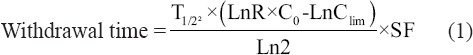


Sample preparation for monitoring drug residues in livers of the goats after the last sampling was prepared as follows: 300 g of the specimen from part of the liver portal vein area were blended in a mortar at 500 g, then the 100 mL of methanol was added and directly shacked for 30 min. Mixtures of samples were centrifuged at 1300× *g* for 15 min, and then, the supernatant was collected and kept in a box of ice at 20°C. The SPE was activated by adding 1 mL of methanol, and then, 3 mL of water was added for chromatography. Then, 1 mL of the supernatant of the samples was added to SPE, and elution was performed at 3 mL in the mobile phase of HPLC. Filtrates from elution of the mobile phase solution were collected in special tubes and drying by nitrogen gas and ready to inject onto the HPLC system.

## Results

**Figure-1:**
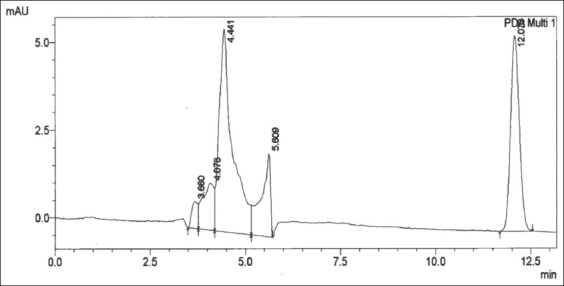
Retention time of clenbuterol at 1.0 µg/mL in goat plasma using the isocratic method high-performance liquid chromatography-photodiode array ultraviolet-visible detector (223 nm) at acetonitrile:water (30:70) containing 0.10% phosphoric acid pH 3.8 in a laboratory room at 23°C-25°C and 46% humidity levels.

**Figure-2:**
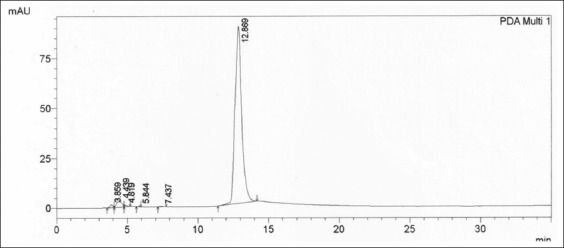
Clenbuterol 0.75 ppm in plasma after being separated with solid-phase extraction C18 1 mg was presented at 12.00 min, and there were no impurity peaks during the monitored 35 min until the stop time.

The results of these observations using spectrophotometer UV-visible showed that the wavelength selected was 223 nm. That wavelength was not intervening to other compounds, and some researchers have already used that wavelength to identify clenbuterol by HPLC in plasma [19]. An analysis with HPLC showed the retention time of clenbuterol from the injection dose regimen was found to be approximately ±12 min (Figures-1 and 2). The low clenbuterol concentration dissolved in the mobile phase but still had an available chromatogram area of approximately 0.001 µg/mL at 33.925. Two concentrations, the upper and lower, were determined to be 0.002 µg/mL, with areas of 67.850, and 0.05 µg/mL, with an area of 1696.258. The regression analysis showed that *R*^2^=0.99 (p<0.05) at Y=−0.000246+33925X. The standard deviation of the triple blank injection signals was 0.2 mm when calculated *with Np-p* at the 20^th^ peak analyte width (0.4 mm, 0.6 mm, and 0.8 mm).

The detection limit was obtained as follows: 
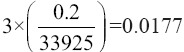
 µg/mL, and the quantitation limit was 0.053 µg/mL. The precision and recovery values of the clenbuterol from the goat plasma are summarized in Table-1. The calibration curve from data in Table-1, at a mean observed concentration (X) versus a mean area chromatogram (Y) by regression analysis was obtained using equation Y=−5379+33941150X (*R*^2^ 0.99; p<0.05). Table-2 shows the calibration curve of the clenbuterol in liver organs free from the residue of the drug as medium matrix biology. The mean of the observed concentration (X) versus mean of the area chromatogram (Y) was obtained with equation Y=−848+33832950X at *R*^2^ 0.99 (p<0.05).

**Table-1:** Calculated concentrations and observed concentrations for intraday precision and recovery analysis in artificial plasma.

Drug	Calculate concentration	Observed concentration (X)	Area chromatogram (Y)	Recovery (%)
Clenbuterol in goat plasma	0.250 mg/mL	0.230	7802790.027	92.000
		0.240	8142041.767	96.000
		0.235	7972415.897	94.000
	Mean±% CV	0.235±2.128	7972415.897±2.128	94.000±2.128
	0.500 mg/mL	0.480	16284083.531	96.000
		0.500	16962587.012	100.000
		0.490	16623335.271	98.000
	Mean±% CV	0.490±2.041	16623335.270±2.041	98.000±2.041
	1.000 mg/mL	1.010	34264425.751	101.000
		0.980	33246670.530	98.000
		0.990	33585922.271	99.000
Mean±% CV		0.993±1.538	33699006.180±1.538	99.330±1.538

Y=−5379+33941150X (*R*^2^ 0.99; p<0.05), CV=Coefficient of variation

**Table-2:** Calculated concentrations and observed concentrations for intraday precision and recovery analysis in liver organs free from the residue of the drug as medium matrix biology.

Drug	Calculate concentration	Observed concentration (X)	Area chromatogram (Y)	Recovery (%)
Clenbuterol in liver organ free from residue of the drug	0.025 mg/mL	0.024	814204.177	96.000
		0.018	610783.232	72.000
		0.020	678113.470	80.000
	Mean±% CV	0.021±14.782	701033.626±14.782	82.667±14.782
	0.050 mg/mL	0.042	1435857.307	84.000
		0.051	1730183.875	102.000
		0.048	1628408.353	96.000
	Mean±% CV	0.047±9.750	1598149.845±9.353	94.000±9.750
	1.000 mg/mL	0.998	32757223.650	99.800
		0.970	33916417.790	97.000
		0.989	33400967.100	98.900
Mean±% CV		0.986±1.450	33358202.850±1.741	98.567±1.450

Y=−848+33832950X (*R*^2^ 0.99; p<0.05), CV=Coefficient of variation

**Figure-3:**
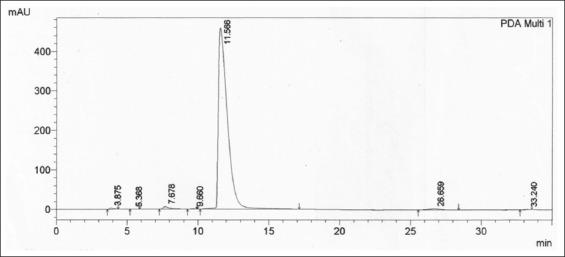
The clenbuterol 0.900 ppm in a liver free from residues of the drug in matrix biology showed impurities from the matrix biology at a retention time of 26.659 min and 33.240 min. However, the clenbuterol was still available at a retention time of 11.566 min.

The average plasma concentration of the clenbuterol determined at various times following intramuscular administration is presented in Table-3 (Figure-3). The concentrations at 5 min of dosing had a mean of 19.233±0.331 µg/mL. The mean of the elimination rate constant (K_el_) from all goats ranged from 5 min to 750 min after dosing was obtained at 0.004/min compared to the elimination half-life measured at 173.25 min. The measured withdrawal times were obtained at 1 day h and 35 min for 1^st^ time SF, but the 2^nd^ time of SF was found at 2.055 days after dosing, and the 3^rd^ time of SF was obtained 3.082 days after dosing.

**Table-3:** The concentration of clenbuterol in goats after giving a single dose of 0.02 mg intravenously.

Time (min)	Concentration of clenbuterol (μg/mL)					

	Goat I (20.5 kg)	Goat II (21.2 kg)	Goat III (20.5)	Goat IV (20.0 kg)	Goat V (21.0 kg)	Mean±SD (20.64±0.47kg)
5	19.021^a^	19.032^b^	19.201^c^	19.101^d^	19.811^e^	19.233±0.331
30	18.700^a^	18.601^b^	18.502^c^	18.021^d^	18.032^e^	18.371±0.322
60	17.202^a^	16.301^b^	18.101^c^	18.011^d^	17.020^e^	17.327±0.746
90	16.051^a^	16.111^b^	17.001^c^	17.902^d^	16.401^e^	16.693±0.773
150	14.001^a^	14.092^b^	15.201^c^	16.151^d^	15.091^e^	14.907±0.888
210	9.502^a^	8.191^b^	7.450^c^	8.201^d^	9.201^e^	8.509±0.834
270	6.001^a^	5.501^b^	6.501^c^	6.801^d^	6.591^e^	6.279±0.525
390	3.801^a^	2.870^b^	3.112^c^	2.812^d^	3.902^e^	3.299±0.518
510	1.701^a^	1.891^b^	1.911^c^	1.861^d^	1.840^e^	1.841±0.083
630	1.530^a^	1.601^b^	1.671^c^	1.651^d^	1.630^e^	1.617±0.055
750	0.901a	0.801^b^	0.851^c^	0.951^d^	0.901^e^	0.881±0.057

Superscripts of the a, b, c, d, e by one-way ANOVA were similar at F=0.01 (p=1.00), Goat-1 Y=21.92.e^−0.004X^, (*R*^2^ =0.983), Goat-2 Y=21.135.e^−0.004X^, (*R*^2^=0.9805), Goat-3 Y=21.986.e−^0.004X^, (*R*^2^=0.978), Goat-4 Y=22.223.e−^0.004X^, (*R*^2^=0.9684)., Goat-5 Y=22.319.e−^0.004X^, (*R*^2^=0.9848)

Figure-3 shows the retention time of clenbuterol in the matrix liver free from drug residues was found at 11.566 min. Monitored residue drugs in the liver are presented in Table-4. The results in Table-4 show the one-sample t-test for all samples was identical.

**Table-4:** Observed 24 h posttreatment residues of the drug in all livers.

Subject sample	Result (μg/mL)
Code liver-1 from subjects 1	0^a^
Code liver-2 from subjects 2	0^b^
Code liver-3 from subjects 3	0^c^
Code liver-4 from subjects 4	0^d^
Code liver-5 from subjects 5	0^e^

Superscript at the same column was identical (p<0.05)

## Discussion

This assessment of withdrawal times depended on the following two aspects: (a) The method for determining the analyte and (b) the sensitivity of the analytical instrument for detecting the analyte. Our method for determining the concentration of clenbuterol in plasma was suitable for the test. The last assessment was measuring the existence of the drug residue in the liver organ.

We found that the retention time chromatogram of the analyte was available for 11-12 min as shown in Figure-1. The chromatogram of the analyte from the goats was never overlayid with other impurity peaks from the biology matrix of the biology compound [19,20]. Figure-2 shows that impurity peaks at more than 12 min until the stop time at 35 min were not found. Figure-3 shows that impurity that peak at a retention time of 26.669 min and 33.240 min were separated from the analyte peak. The impurities peak in picture 4 was possibly part of the matrix liver compound and not be derived from the metabolite clenbuterol. The international requirements mentioned that the conditions of the resolution (α) should not be the same with 1 or α≠1 [20]. The correlation-regression analysis of clenbuterol at 0.25 µg/mL-1.00 µg/mL from the plasma matrix was linear at *R*^2^ (p<0.05) with recovery between 94.000±2.128% and 99.330±1.538% of each concentrate [21]. Then, the *R*^2^ of the correlation-regression for analysis of clenbuterol in the liver matrix as a blank compound was 0.99 at p<0.05 (Table-2). The recovery of the liver matrix compound ranged from 82.667±14.782% to 98.567±1.450%. The results from HPLC, shown in Table-1, indicated that the calculated concentrations between the observed concentration were recommended; thus, concentrations lower than 0.235 µg/mL or higher than 0.933 µg/mL are not recommended as precise and accurate (p<0.05). The residue clenbuterol in the liver matrix within the range of observed concentrations, described in Table-2, is recommended, but concentrations lower than 0.021 µg/mL or higher than 0.986 µg/mL, are not recommended as precise and accurate (p<0.05). Precise and accurate analysis in matrix biology (plasma or organ matrix biology) was available approximately at more than 20% of the coefficient of variation (CV), and approximate recovery was in the range 80-120%. At low concentrations, there were large CV values, so at high concentration values, there were small CV values [16,20,22]. The sensitivity of the test, according to measurements of the detection limit and quantification limit, was similar to that of a study reported in 2016 [23]. The results of this research indicated that the HPLC method for determining analytes in matrix biology (plasma and matrix livers free from drug residues) were suitable for the test.

**Figure-4:**
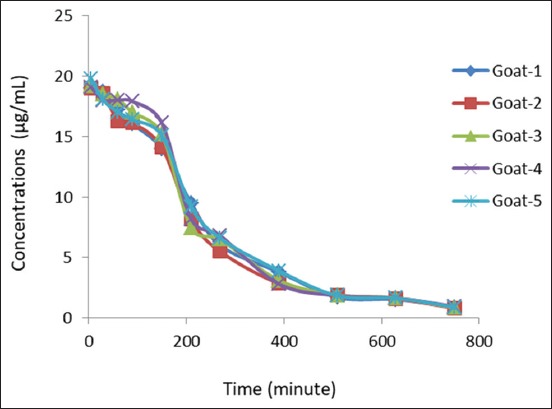
Concentrations of clenbuterol (µg/mL) versus time average (min) for goats administered a single dose intravenously at 0.02 mg.

Profile concentrations of clenbuterol in the plasma were apparent in two intravascular compartment models as follows: In the central compartment, the clenbuterol is rapidly distributed throughout the body, and the drug is then gradually eliminated from the body as a peripheral compartment (*R*^2^ 0.97-0.98). 5 min after dosing, the maximum concentration obtained was 19.232±0.331 µg/mL with elimination continuing rapidly as presented in Figure-4 [24]. A decreased mean concentration level of 3.84% resulted from the dose and produces initial concentration levels of less than the dose after 5 min posttreatment. These levels occurred because of the drug spreading instantly throughout the body through the bloodstream after intravenous administration [25,26]. Drugs with strong macromolecule-drug bonds tend to decrease rapidly after the initial administration of drugs such as clenbuterol as an analog [17]. Table-2 shows that the elimination half-life was obtained at 2 h and 48 min, which was less time than for other reported research. Research reports have described the elimination half-life occurring on average at 16 h-105 h in cattle that were administered the drug at 8 µg/kg BW twice a day. These differences occur because of differences between species and dose regimens.

Observations of the data available for clenbuterol in goats indicated that the drug had a rapid onset of action as a type of short-acting β_2_-agonist. The elimination phase constant indicated that clenbuterol is eliminated from the body at a slow elimination phase constant. 2-3 days are needed for the drug to disappear from the body after the administration of 0.625 mL of an injection dose regimen. Commonly, the usual single dose needed to obtain a therapeutic effect is 2.5 mL for each 100 kg of body weight. If the animal is given a double dose, there is a risk that the body will have 2 times the amount of drug residue. Thus, a longer time is needed to ensure the absence of a drug residue. Therefore, animals can be safely slaughtered after a drug has passed from the body after treatment. Further, evidence indicated that at 24 h posttreatment, drug residue on the samples was not found in all of the organs in the liver (Table-4, p<0.05). The results showed that clenbuterol was entirely removed from the body of the goat after 24 h. In theory, there is clearance of the clenbuterol. Compared to other researchers, at a dose of 6 µg/kg of body mass given orally once daily over 21 days, male goats (30 kg body weight) had liver residues 0.5-200 µg/L. The mean concentrations at 24 h showed liver residues between 0.023 µg/L and 9.524 µg/L [22]. By comparison with our research as theoretically calculated, the individual goats were divided 9.10^3^ times, and we obtained concentrations of 0.000 µg/L for liver residues. Clenbuterol was metabolized in the liver by the conjugation process, and the metabolite will be accumulated in the liver [17]. The clenbuterol metabolite will be easily found in the liver when there is a sufficient amount of drugs. However, other organs will accumulate a proportional amount as follows: Lung 25%, muscle 10%, kidney 50%, and heart 50%, fat 10%, eyes 1%, plasma 50%, urine 50%, and bile 10% [22]. In this research, the observed amounts were obtained from the plasma and clarification in liver organs. This finding was in accordance with the proportion of the spread of clenbuterol in the body.

Therefore, the use of clenbuterol as a treatment should proceed with logic and responsible application to the body [12,27,28]. If the calculation of withdrawal times was performed using the new method for calculating withdrawal times by substitute of Co in equation 1, with doses administered as accumulate drugs, the results would be satisfactory with no drug residues in all organs [20].

The new method for the design of mathematical models was explained in equation 2. Prediction using the equation 2 for determination of withdrawal times as describe at below is easy to apply and suitable for all species or all of the veterinary drugs, although they need data on the elimination half-life for all of the drugs. The difference between equation 1 and equation 2 is the highest concentration of the drug in the body, as follows: Equation 1, the highest concentration at attributes Co; in equation 2, the highest concentration was the the highest concentration was the


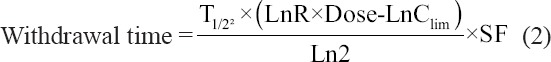


In many countries, the use of clenbuterol requires a veterinarian prescription because it is a hazardous drug and is very easily abused. However, recommendations for the legal use of clenbuterol are safe, especially for animals that will be consumed by humans [22,29-31]. These animals include not only goat species but also other polygastric species, including sheep and cows. This study did not specify residual clenbuterol levels in the animal body but did determine a safe condition that was free of the clenbuterol residues after being given in therapeutic doses [32].

## Conclusion

Evidence showed a safe withdrawal time of 0.02 mg clenbuterol in a formula of 0.625 mL of an injection dose regimen in goats for 2 days, 13 h, and 12 min at the 2^nd^ SF or 3 days, 1 h, and 58 min at the 3^rd^ SF. These research results are recommended for application to other polygastric species, such as cows and sheep. However, the administration of double doses must be advantageous in research, especially at level concentrations of the drug in the plasma and residues of the drug in the liver.

## Authors’ Contributions

LM was a research coordinator and drafted the manuscript, BH assessed the validation method, and TIR carried out a health analysis of the goats. All of the authors read and approved the final manuscript.
